# Spatio-temporal distribution and evolution of the Tujia traditional settlements in China

**DOI:** 10.1371/journal.pone.0299073

**Published:** 2024-03-11

**Authors:** Yunzhang Li, Jianjun Du, Disi Ran, Cao Yi

**Affiliations:** 1 College of Architecture and Environment, Sichuan University, Chengdu, China; 2 School of Architecture and Civil Engineering, Xihua University, Chengdu, China; Al Mansour University College-Baghdad-Iraq, IRAQ

## Abstract

The rapid modernization in China has aggravated the reduction of the traditional settlements and aroused concern about the protection and research. This study aims to examine the spatial-temporal variations Tujia traditional settlements in China and to delineate the driving mechanism of the settlement distribution. Previous studies have focused on the characteristics of settlements in provincial or smaller areas, providing lacked information regarding spatial distribution heterogeneity of Tujia traditional settlements in China. In this study, the spatial heterogeneity and influence factors of the distribution of traditional Tujia settlements were examined using the GIS platform and statistical methods. The results reveal that the spatial distribution of settlements exhibits clustering with the pattern of “scattered distribution in a large region, while concentrated in small areas”. The settlements were generally built in low hills, gentle slopes, sunny slopes and low-relief terrain areas, with elevation, relief degree of land surface (RDLS), slope and aspect were the key factors affecting the distribution. In Song, Yuan, Ming and Qing dynasties, settlements showed significant clustering all through, though the location and number of clustering center kept changing. In this process, the history of the Tujia chieftain and the transportation and marketing lines of Sichuan salt had a profound influence on the historical evolution of the settlement.

## 1. Introduction

The traditional settlement is the bright pearl of Chinese traditional farming culture, the preservation place of human material and cultural heritage, and the fossil garden of intangible cultural [1]. Recently, with the rapid urbanization and beautiful rural construction, traditional settlements have been swallowed by modernization, renovated or replaced, and the existing number has been decreasing rapidly, which is facing the downfall or extinction. Therefore, it is of great practical significance to increase the attention to Chinese traditional settlements and the research on it.

In recent years, geographical datas and methods have been extensively used to study the spatio-temporal village distribution, and interactions between humans and the environment [2–4]. Scholars mostly have used quantitative geography and Geographic Information System (GIS) technology to examine the spatial distribution rules and driving mechanism of traditional villages in different areas, topography, transportation and economy have become important factors affecting the distribution and protection of traditional settlements [4–6]. While some others also used the analytic hierarchy process (AHP) [7], a qualitative description and more subjective method to study the settlement by expert scoring. With the development and application of GIS technology and statistical software, attempts have been made to settlement distribution with a broader natural factors analysis, using remote sensing, GIS, models and other computer software techniques to analyze the spatial characteristics, evolutionary mechanism and optimization of settlements [8, 9], and offering a novel approach to the study of human-earth environment interactions and cultural development from the standpoint of spatial analysis. These studies demonstrated that a combination of geographical information and GIS analysis is useful for studying spatio-temporal and evolution of settlements distribution. However, most recent studies have been focused on settlement patterns, landscape, architecture or protection and development [10–12], which mainly involved the mesoscopic and microscopic aspects, and the macroscopic concentrated on the provincial scale. Systematic, quantitative analysis on a broader regional scale has been lacking.

As an important part of Chinese traditional villages, the Tujia traditional settlement embodies the profound historical, cultural, and humanistic legacy, and remains the living cultural treasures of the ethnic group itself. Until now, studies on it have primarily focused on specific areas settlement patterns, dwelling characteristics and Tujia culture [13–15]. Comprehensive studies on the traditional Tujia settlements in Hunan, Hubei, Guizhou provinces, and Chongqing municipality, especially on the spatio-temporal distribution and evolution, and the grasp of regional integrity [16] are rare. This paper seeks to contribute to this area. The study describes spatio-temporal patterns and evolution characteristics of Tujia traditional settlements, by using GIS platform to build a spatial geographic database and combines quantitative and qualitative methods based on literature retrieval and field investigation. The reasons for the formation and development of settlements are discussed from a broader regional and cultural perspective, with a comprehensive analysis of spatio-temporal and evolution rules and cultural connotations, and hopes to provide reference for the overall protection and development of Tujia traditional settlements. The methods adopted in the research can be applied into the study of other settlements.

## 2. Study area

**Fig 1:**
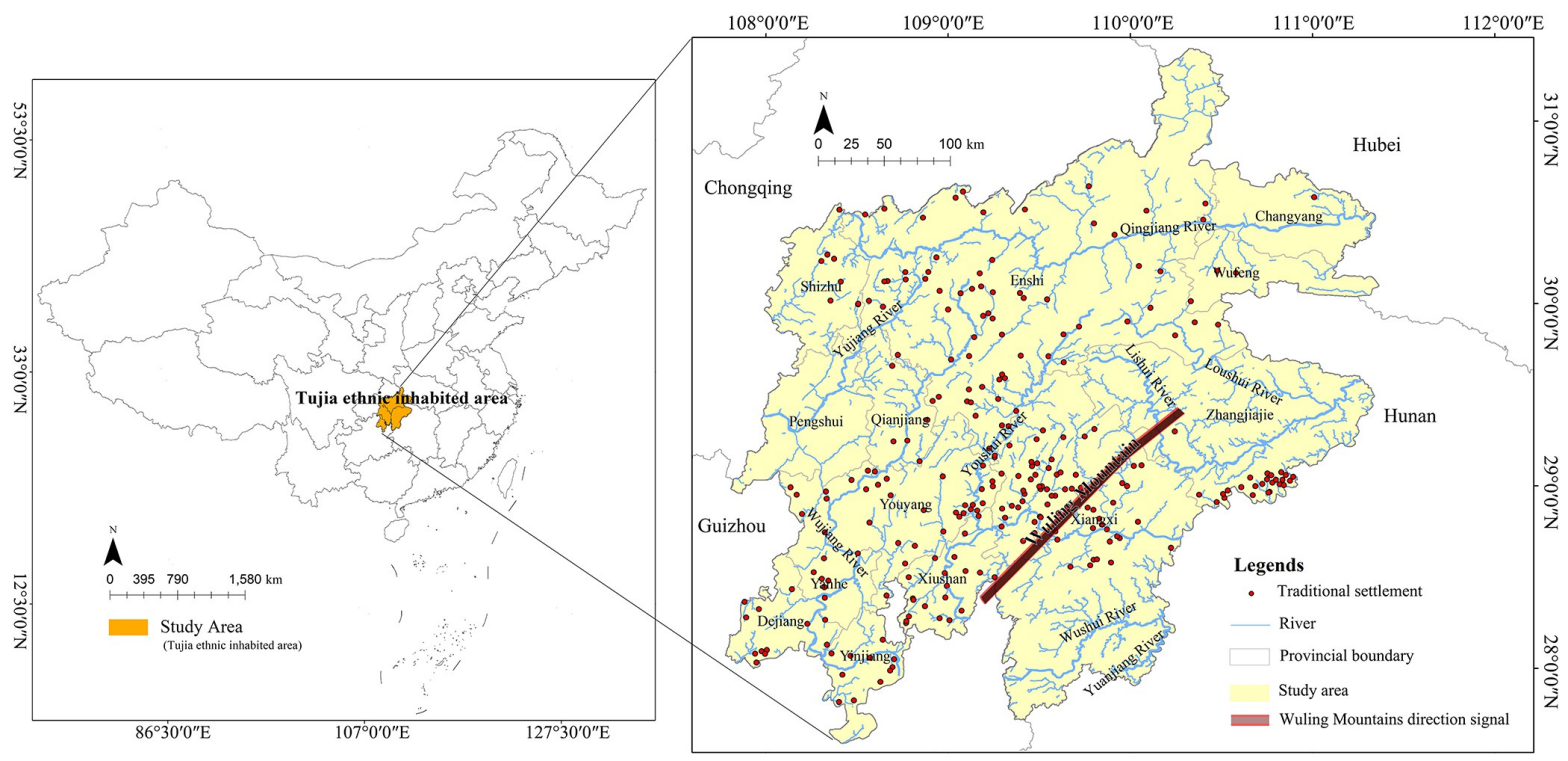


The Tujia people have lived for generations in Wuling Mountains area, a region bordering of Hunan, Hubei, Guizhou provinces, and Chongqing municipality, ranging from 107.78°00′E to 111.36°00′E and 27.59°00′N to 31.39°00′N (Fig 1). In general, the Tujia settlement area centers on the eastern Wuling Mountains and the Qingjiang River basin, reaching the Fanjing Mountain and the Wujiang River in Guizhou to the west, the Yiling (now Yidu in Hubei) and the Jianghan Plain to the east, the Wushan Mountain and the Yangtze River to the north, and the Lishui and Yuanjiang River to the south, covering an area of about 90,000 km^2^. According to the China Statistical Yearbook-2021, the population of Tujia in China is 9,587,732, with about 90% of the total living in the Wuling Mountains and the rest being scattered in Sichuan, Zhejiang, Guangdong, etc. As this paper focuses on the traditional settlement areas where Tujia people live in compact communities, the scattered areas are not discussed here.

**Fig 2:**
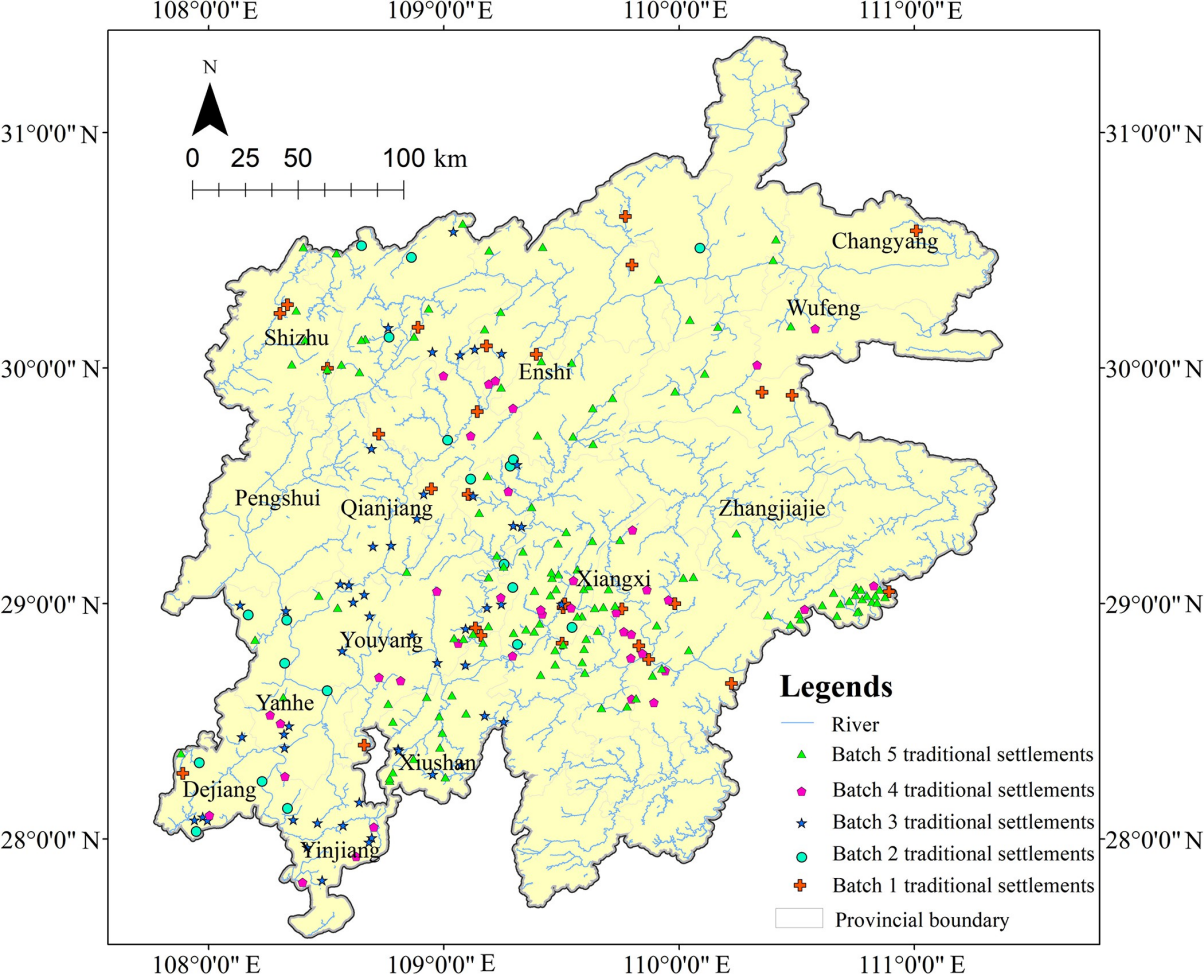


The traditional Tujia settlements are mainly listed in the five batches of Traditional Chinese Villages Catalog by the Ministry of Housing and Urban-Rural Development (MOHURD) and other departments up to 2021, as well as the National Famous Historical and Cultural Villages published by MOHURD and the National Cultural Heritage Administration of China, totaling 265 settlements (Fig 2). As the settlements are concentrated in the Wuling Mountains area where numerous mountains lead to varying geographical conditions and natural environments, the spatial distribution of settlements is quite diverse.

## 3. Materials and methods

### 3.1 Data sources

To pursue accuracy and systematicity of data analysis, the data in this study were obtained from the official authoritative website and calibrated by ArcGIS10.8, with geographic coordinates of WGS_1984. Details on the datas used for this study are presented in Table 1, the information were imported into GIS platform to establish a spatial geographic database, and each data was overlaid analysis (OA)^Ⅰ^ and visualized.

**Table 1:** Data sources and processing.

Data categories	Data type	Data sources	Data processing
Settlement point	Latitude and longitude	Geospatial Data Cloud (https://www.gscloud.cn/search)	The settlement locations were located by Google Earth and visualized in GIS.
Elevation	National elevation data (with 30m resolution)	Resource and Environment Science and Data Center of Geographic Sciences and Resources Research, CAS (https://www.resdc.cn/)	By using the “clip” or “extract by mask” tools in GIS, elevation datas were obtained to extract and analyze slope and aspect.
River and Road	Distribution map of river and road in China		By applying the “clip” tool in GIS, river and road datas were obtained and then analyzed by the density analysis tool.
precipitation	1901–2020 Monthly precipitation dataset with 1km resolution in China	China Meteorological Data Service Centre (http://data.cma.cn/)	By using the “clip” or “raster calculator” tools in GIS, the monthly average precipitation datas were obtained.
Ancient Sichuan salt route	Distribution map of the salt route	Sichuan Salt Trail: Architecture and Villages in the Field of Vision of the Cultural Route [17]	The scanned map was geographically aligned in GIS to trace the salt route, and to OA of the route and settlements.
Period of settlements establishment	Information on Song, Yuan, Ming and Qing dynasties	Records of counties and villages in the study area	OA and density analysis were performed in GIS.
Tujia chieftain resident	Distribution map of Tujia chieftain	Historical Atlas of China [18]	The scanned map was geographically aligned in GIS to trace the chieftain resident, and to density analysis of the resident and settlements.
The basemap	Shapefile	OpenStreetMap (https://www.openstreetmap.org/copyright)	Import the data to the GIS for projection processing.

### 3.2 Methods

**Fig 3:**
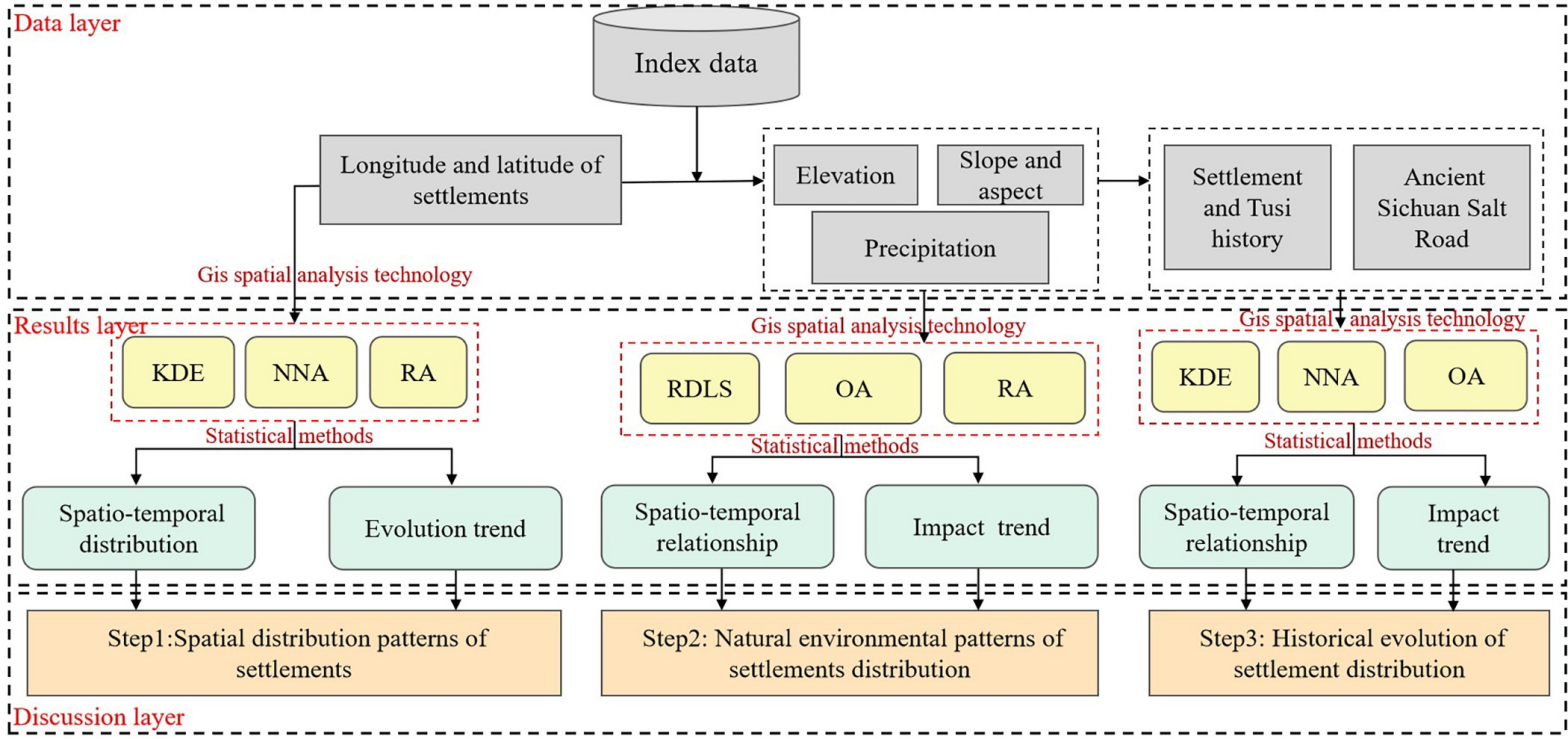


Fig 3 outlines the research structure and technical framework of this study. It mainly applied GIS and statistical software, and kernel density estimation analysis (KDE), nearest-neighbor analysis (NNA), OA, relief degree of land surface (RDLS) and road accessibility (RA) were used to study the spatio-temporal and evolution patterns of the Tujia traditional settlement distribution.

#### 3.2.1 Kernel density estimation analysis

KDE is the density of analysis elements in its surrounding neighborhood, which can show the concentration and dispersion in space. The degree of density can reflect the probability of an event occurrence that the more dense the probability of occurrence is higher, the more sparse the occurrence is lower [19]. The spatial density features of traditional settlements were obtained with this tool of GIS.

#### 3.2.2 Nearest-neighbour analysis

The nearest neighbor distance measures the distance between each feature centroid and its nearest neighbor’s centroid location, and the Average Nearest Neighbor (ANN) distance is the average of all nearest neighbor distances. KDE provides the average value of point density in a certain area but cannot show cluster features whereas ANN can be used to measure the features in a region. The calculation formulas are as follows:

$$ANN=\frac{\bar{D_{O}}}{\bar{D_{E}}}$$ (1)

$$\bar{D_{O}}=\frac{\sum_{i=1}^{n}d_{i}}{n}$$ (2)

DE¯=0.5nA (3)

Where D_O_ is the observed mean distance between each point and its nearest neighbour, and D_E_ is the expected mean distance for the point given in a random pattern. D_i_ equals the distance between point I and its nearest neighbor, n is the total number of points and A is the area of a minimum enclosure. According to the criteria, the value of the ANN ratio indicates whether points are clustered (< 1), random (= 1) or dispersed (> 1) in space [20, 21]. which is calculated based on the average distance from each point to its nearest neighbor.

The ANN tool in GIS calculates not only the ANN ratio of settlements, but also the p-value representing probability and the z-score representing standard deviation. The p-value and z-score are measures of statistical significance used to ascertain whether to reject the null hypothesis (i.e., the hypothesis of complete spatial randomness). The smaller that the p-value is and the larger the absolute value of the z-score is, the less likely the observed spatial distribution is the result of a random process. If the p-value is less than 0.01 and the absolute value of the z-score is larger than 2.58 (i.e., < -2.58 or > +2.58), the confidence level would be over 99% [22].

#### 3.2.3 Relief degree of land surface

RDLS can reflect the relative elevation difference of the ground, and is a quantitative index to describe the landform [23], its formula is as follows:

RDLS=ALT/1000+[max(H)‐min(H)]×[1‐P(A)/A]/500 (4)

Where ALT is the average altitude within a certain area centered on a raster cell, max(H) and min(H) are the highest and lowest altitudes (m) of a region respectively, P(A) is the flat area (km^2^), and A is the total area. The terrain with a slope less than or equal to 5° is flat, and its area is P(A). According to the research of the Sustainable Development Strategy Study Group of the Chinese Academy of Sciences (CAS), 500 meters is regarded as the base elevation. So, when RDLS is less than 1, it indicates the topographic relief is below that of the base elevation, and if RDLS equals 1, the relief equal to the base, and hence when RDLS equals k, it indicates the topographic relief k times the relief of base elevation [24]. According to the indicators of the basic landforms in China, the RDLS of the Tujia settlement area was classified, namely, 0–1.0 as low relief area; 1.0–2.0 as medium relief area, with 1.0–1.5 as medium-low relief area, 1.5–2.0 as medium-high relief area; 2.0–3.0 as high relief area.

#### 3.2.4 Road accessibility

RA measures the cost of time between points by converting the distance traveled by different modes of transport into time, it is influenced by two variables: road network and travel speed. The road network structure of this study includes railways, highways, national highways, provincial highways, county roads, and others. Road speeds are set according to the road mileage and speed standards of categories in China 2010 and the Technical Standards of Highway Engineering (JTG B01-2003) as well as relevant studies [25] (Table 2). In this paper, the Tujia settlement area was divided into several homogeneous networks of 1km*1km, assuming that the accessibility within the network was the same, which means that each network was regarded as a homogeneous and accessible point [26]. The formula for determining the accessibility of any point in the region to the settlement is A_i_ = min(T_ij_), where i is any point within the research area, T_ij_ is the minimum time cost of the shortest route to settlement j through the traffic network from settlement i, and A_i_ is the RA of settlement i. A_i_ can be used as a criterion for a settlement’s accessibility, that the smaller the A_i_, the better the RA of the settlement, and the worse the opposite.

**Table 2:** Conversion of spatial distance and time cost of road network.

Road classification	Railway	Highway	National highway	Provincial road	County road	Others
Velocity (v)(km/h)	100	120	80	60	40	20
Time cost (t)(min)	0.6	0.5	0.75	1	1.5	3
Formula	Cos/t = 60/v					

## 4. Results

### 4.1 Spatial density

**Fig 4:**
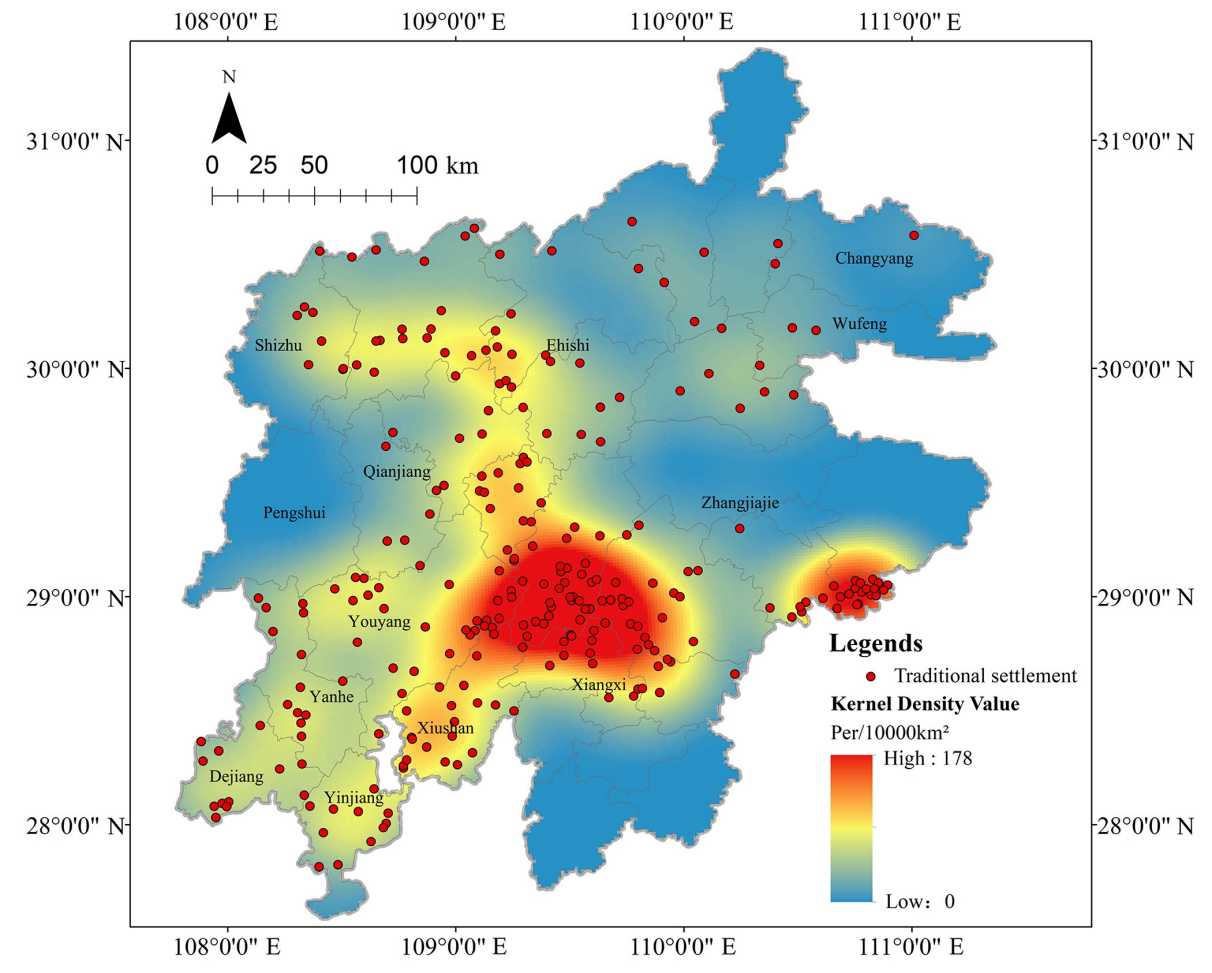


The density analysis results (Fig 4) reveal that the Tujia traditional settlements have mainly concentrated in the southwest of the central Wuling Mountains area, with significant spatial differences and the overall spatial distribution patterns of “two belts, one core, and multiple nodes”. To be specific, the “two belts” are the north-south and the east-southwest development belt. The “one core” is the central core area of Wuling Mountains, with a density range of 99–178 units per 10,000 km^2^. “multiple nodes” means the Enshi Tujia and Miao Autonomous Prefecture (Laifeng, Xianfeng, Lichuan) in southwest Hubei, the Xiushan in Chongqing, the Yanhe and Dejiang regions in Guizhou, etc., with a density range of 39–99 units per 10,000 km^2^. The core area of the spatial distribution of settlements is obvious, and showing a pattern of “scattered distribution in a large region, while concentrated in small areas”, with an average distribution density of 34 units per 10,000 km^2^. While the Tujia traditional settlements left in Changyang, Wufeng, and Pengshui counties, as well as the counties and cities in the southern part of Xiangxi Tujia and Miao Autonomous Prefecture, are relatively few, especially in southern Xiangxi Tujia and Miao Autonomous Prefecture, where the spatial density is extremely sparse. The characteristics and driving mechanisms of the revealed spatial distribution patterns are further investigated in the following.

### 4.2 Nearest-neighbour analysis

**Fig 5:**
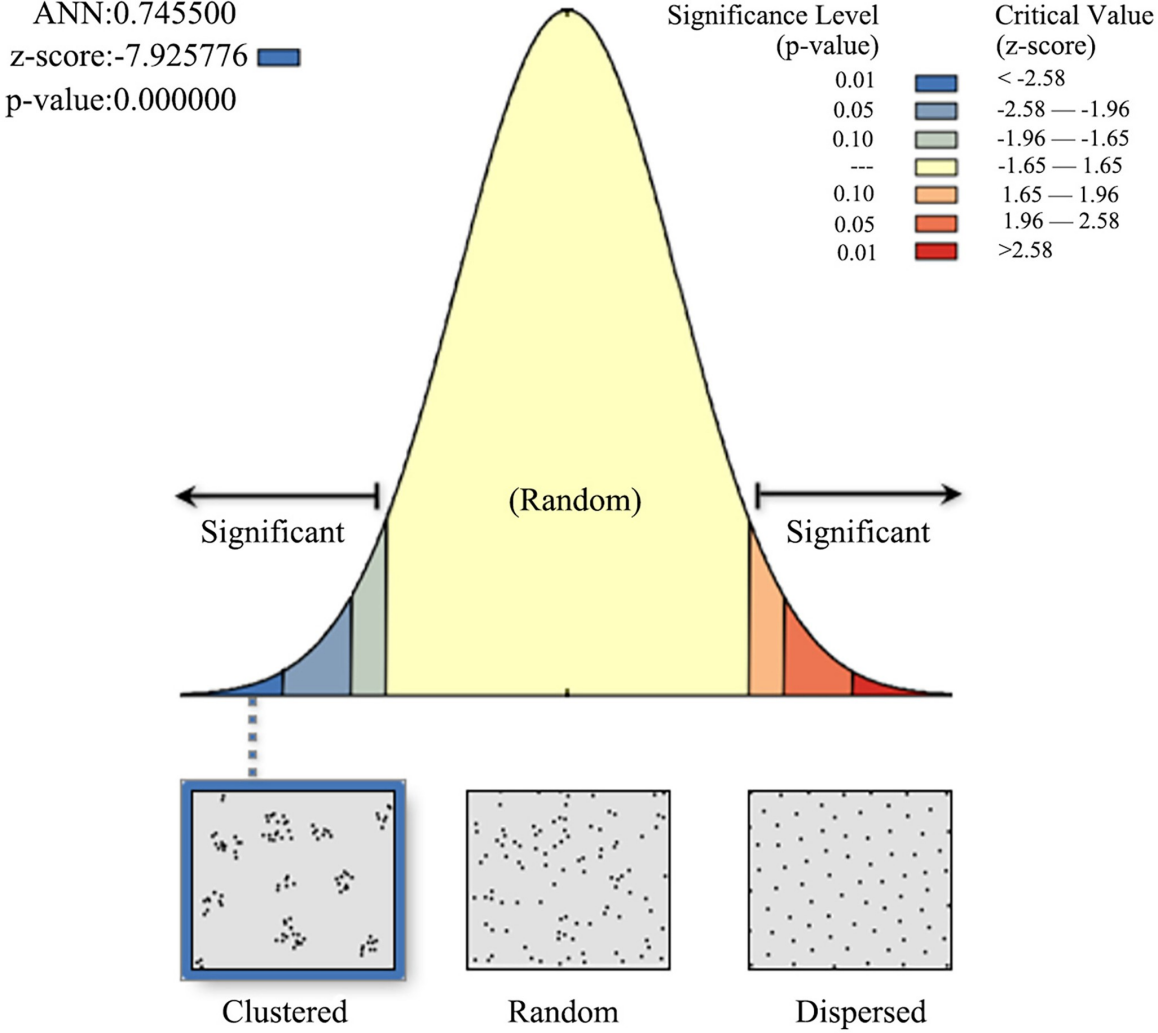


Using the ANN tool in GIS, the ANN value, p-value and z-score of traditional settlements were calculated (Table 3 and Fig 5). According to their critical values, the Tujia traditional settlements exhibit clustering with the confidence levels over 99%. According to the search of county and village chronicles and the on-site investigation, the Tujia traditional settlements were mainly built in the Song, Yuan, Ming and Qing dynasties. Among the settlements of all dynasties, the settlements in the Ming Dynasty have the highest level of clustering with the highest confidence level. The Qing Dynasty comes second, followed by the Song and Yuan dynasties. The reasons for the differences in the number and clustering degree of settlements in different dynasties are further investigated in the following.

**Table 3:** Results of ANN of the settlements of each Dynasty.

Dynasties	Number of settlements	D_O_ /km	D_E_ /km	ANN	p-value	z-score	Distribution type
Song (Before 1279)	13	29.066	34.988	0.8307	0.243	-1.168	Clustered
Yuan (From 1279 to 1368)	31	19.656	23.277	0.8444	0.097	-1.658	Clustered
Ming (From 1368 to 1644)	113	10.091	13.850	0.7285	0.000	-5.520	Clustered
Qing (From 1644 to 1912)	265	7.017	9.412	0.7455	0.000	-7.926	Clustered

### 4.3 Road accessibility

**Fig 6:**
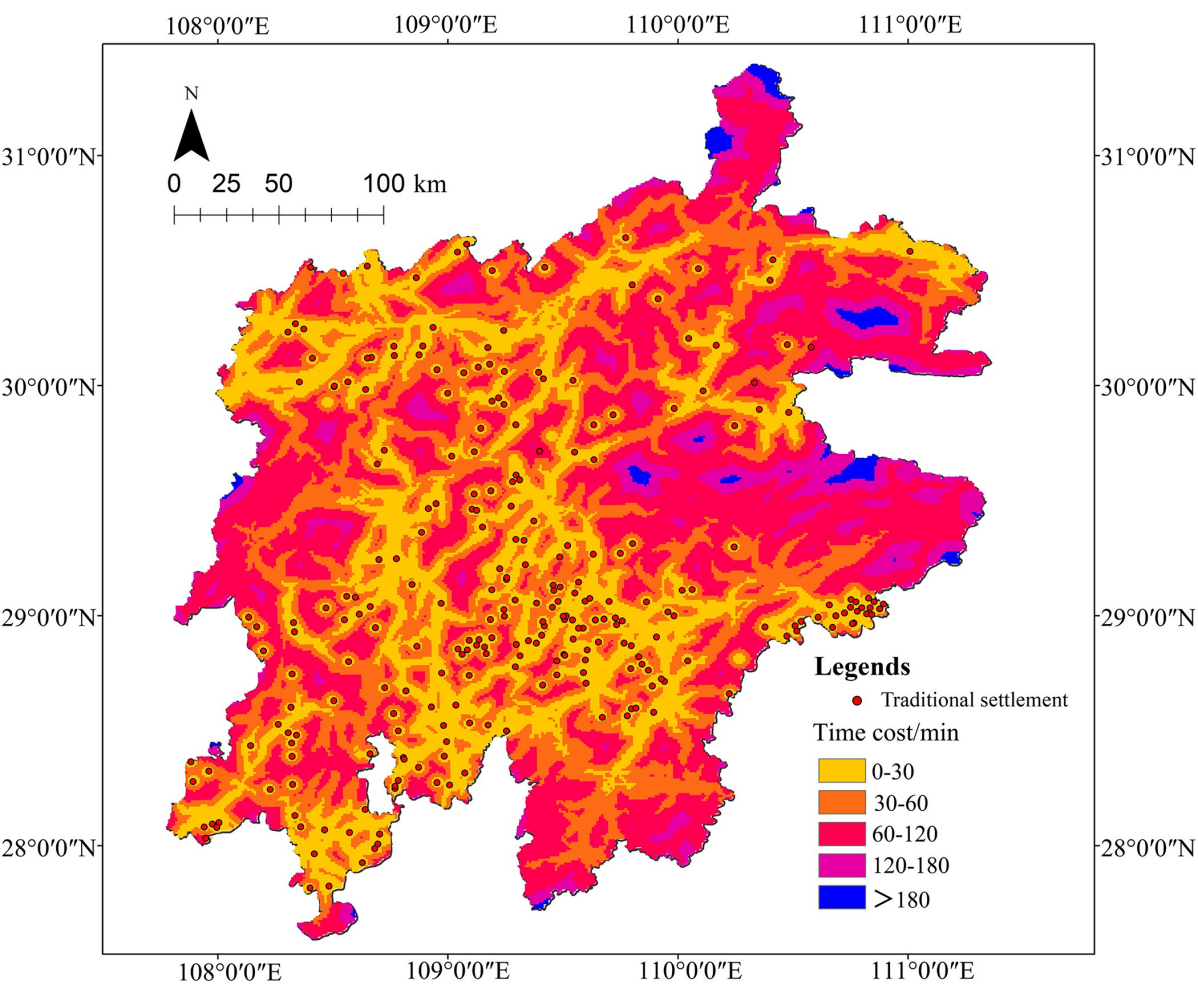


**Fig 7:**
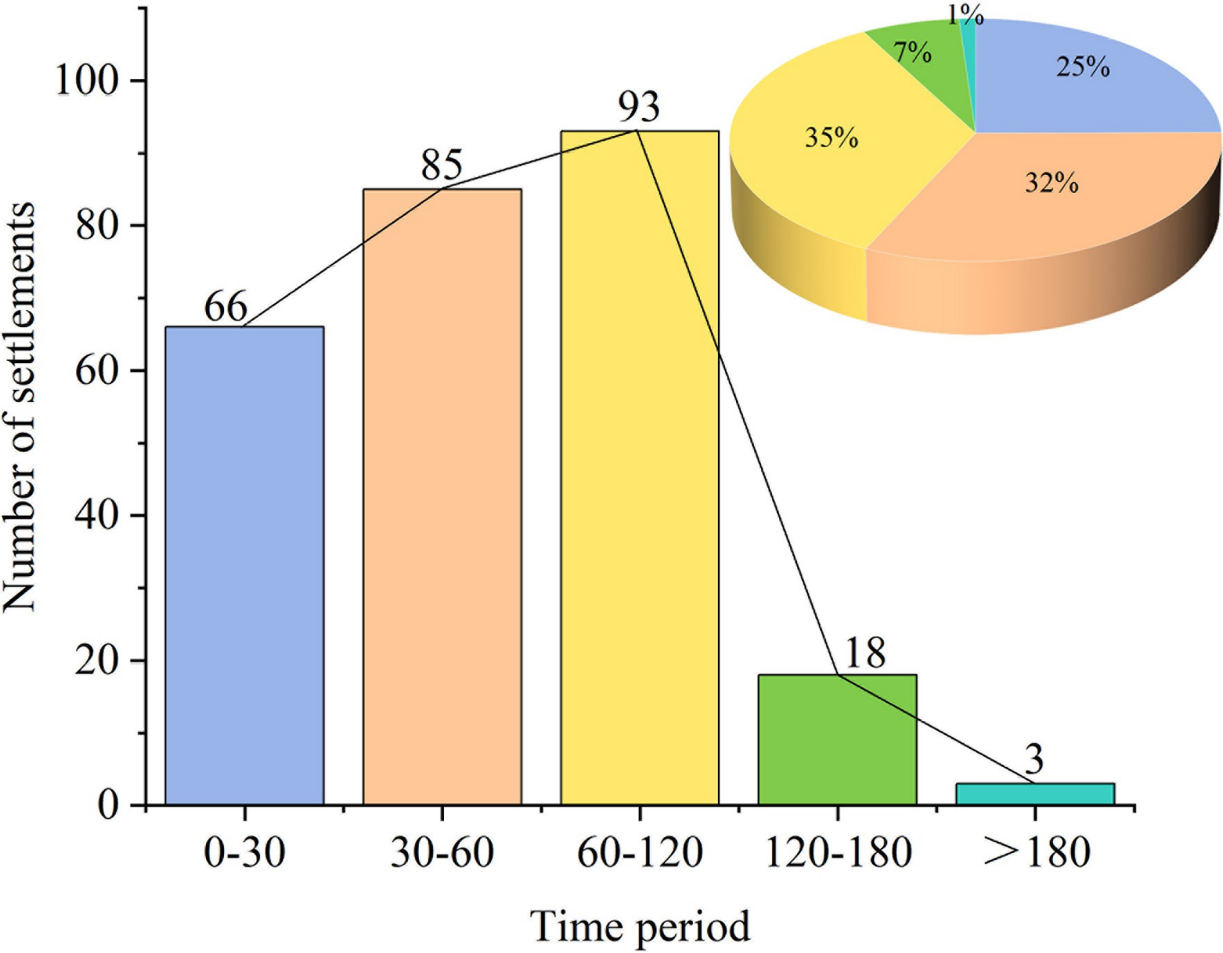


According to the time-cost analysis, the shortest time required for each settlement to reach any homogeneous network (1km*1km) within the research range was calculated based on the settlement as the distance source point and the road network. The number of effective rasters within the area is 78,495, and the RA of the settlements is shown in Fig 6. The accessibility of these settlements was divided into five periods, namely, 0–30 min, 30–60 min, 60–120 min, 120–180 min, and above 180 min [27], and the proportion of each period’s accessibility in the research area was calculated respectively to analyze the spatial distribution of each period (Fig 7). The statistical results suggest that the average reachable time of the settlement is 56.12 min, with 57% of them within 60 min and 92% within 120 min of the studied area, indicating an overall rather good accessibility of these settlements. From the spatial distribution of each period, the accessible time ranges from 0–30 min (25%), 30–60 min (32%), 60–120 min (35%), 120–180 min (7%), and over 180 min (1%). It could be inferred that with 43% of the periods above 60min and 8% above 120min, the proportion of settlements with poor accessibility remains relatively small. The frequency of the distribution tends to increase as time goes up within 120 min.

## 5. Discussion

### 5.1 Spatial distribution patterns of settlements

**Fig 8:**
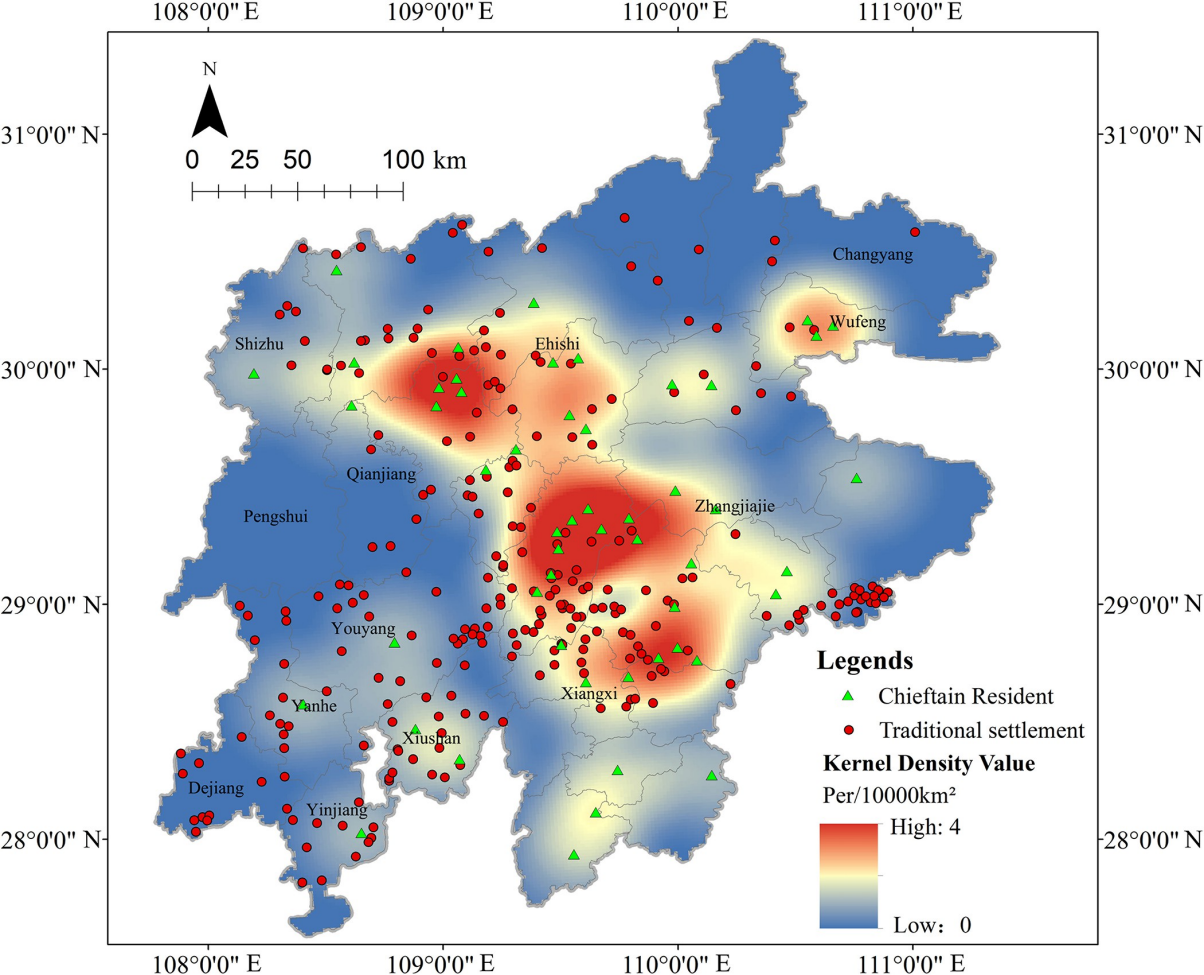


**Fig 9:**
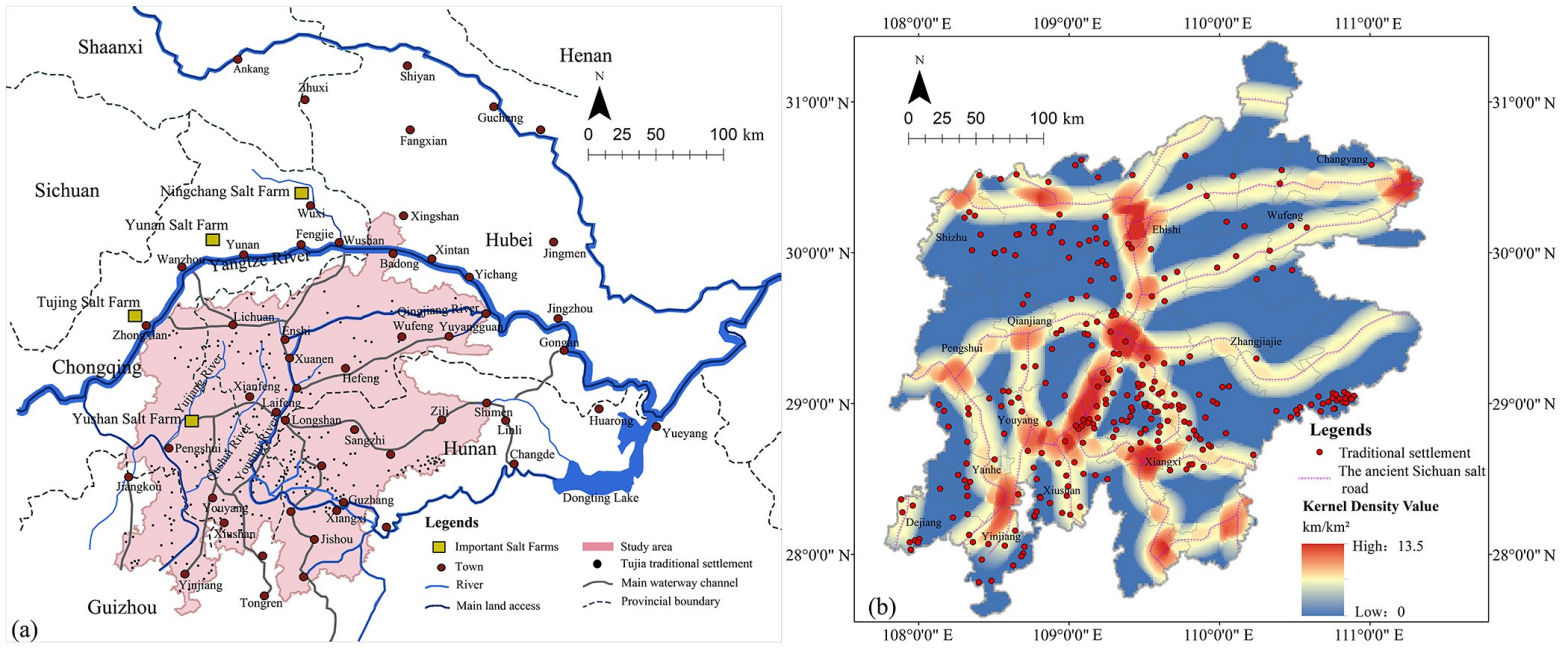


The spatial distribution patterns of Tujia traditional settlements can reveal important information on national history, culture and settlement development. Fig 4 shows that Tujia traditional settlements mainly live in the southwest of the central part of Wuling Mountains area, and are sparsely in the south and east. It is found that this’s related to the distribution of Tujia chieftains and the transportation and marketing routes of Sichuan salt through literature search, and this inference is supported by cademic research [16, 17]. From Yuan and Ming dynasties to the early Qing Dynasty, the imperial court implemented the chieftain system in the Tujia area, built the chieftain’s residence to occupy the military forts (Fig 8). The chieftain system was the “hotbed” for the formation of the Tujia minority, that laying the foundation for the Tujia to become a single minority nation and the settlement. After the Qing Dynasty “bureaucratization of native officers”, that the chieftain system and the territorial economy were disintegrated, and the ban of “native people were not allowed to leave the territory and no Han Chinese were permitted to enter” [28] was cancelled. And a large number of Han people poured into, which greatly promoted the development of agricultural economy and the settlements gradually expanded along the chieftain’s residence. Fig 8 shows there are few chieftain residents in the south and east of Wuling Mountains area, and other ethnic minorities were gathered there, so there were few Tujia settlements left in this area. Salt trade promoted the development of settlements in ancient times. The area covered by Sichuan salt was mainly the middle and upper reaches of the Yangtze River, including where Hunan, Hubei, Guizhou provinces, and Chongqing municipality meet, that is, the Wuling Mountains. As remote and traffic inconvenience, besides shipping by river, salt was mostly transported by humans and horses, thus many bazaars and post stations flourished along the route. Later, the settlements expanded around it, many ancient dwelling settlements with local architectural styles had been built. From Figs 8 and 9, the density of the chieftain resident ranges from 0 to 4 units per 10,000 km^2^, with a maximum of 4, and the density of the ancient Sichuan salt road ranges from 0 to 13.5km/km^2^, with a maximum of 13.5. It can be seen that salt roads and chieftain resident were dense in the central Wuling Mountains area, with well-developed river and road network, while were sparse in the south and east, which is consistent with the distribution of Tujia traditional settlements, verifies the above analysis.

From Figs 6 and 7, it can be concluded that the accessibility of the settlements is mostly at a medium level. However, due to the uneven spatial distribution and the higher altitudes in the mountains of the northeastern and western regions, cars are the main means of transport, and there are mostly provincial highways and county roads, which requires a longer time to travel the same distance, making the settlements relatively difficult to reach. This situation also needs to be improved for further development.

### 5.2 Natural environmental patterns of settlements distribution

#### 5.2.1 Terrain analysis

**Fig 10:**
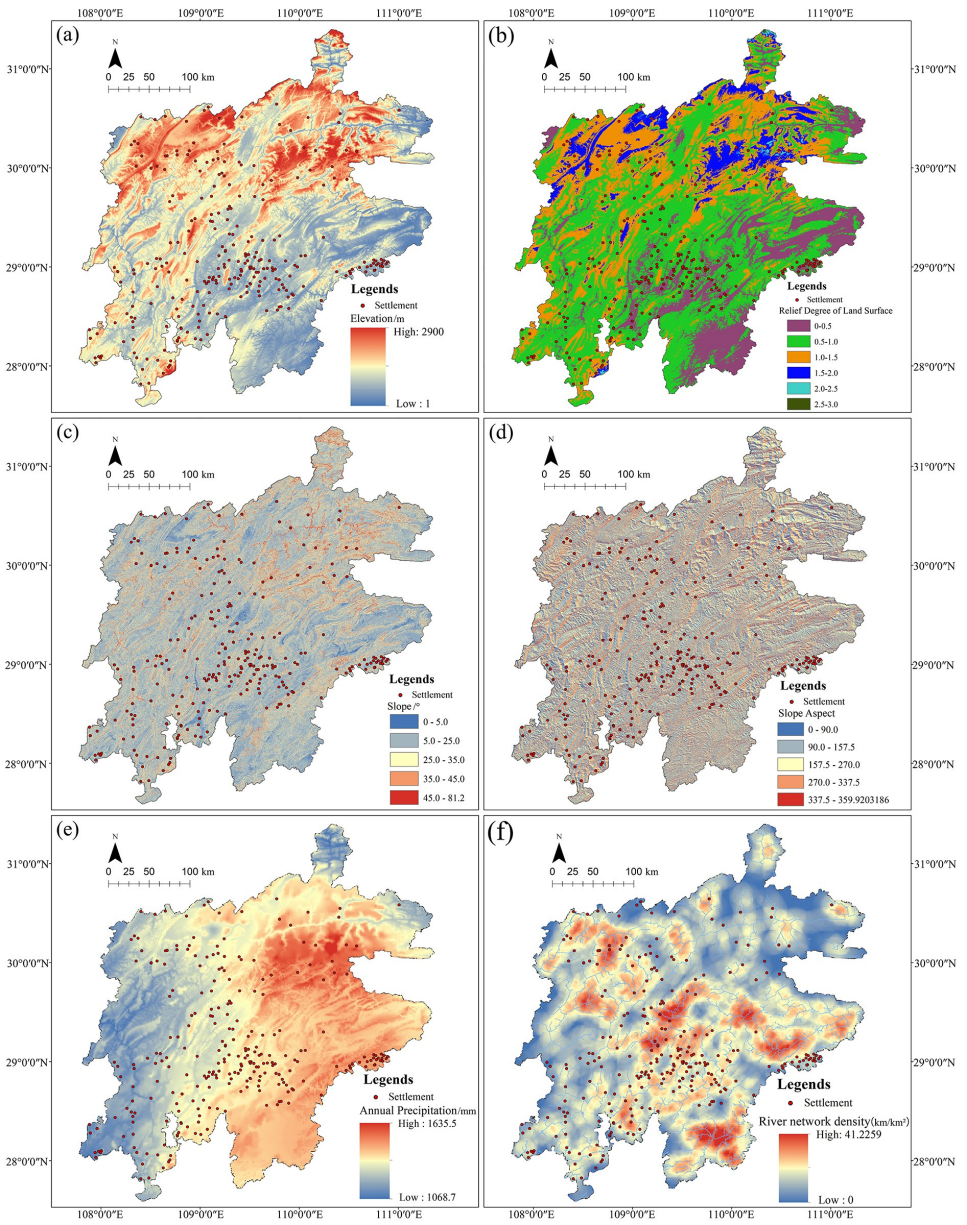


As a basis for the formation and development of settlements, natural geographical factors influence the spatial distribution of Tujia traditional settlements in the Wuling Mountains(Fig 10). The terrain are major factors, which not only provides space for the establishment and development of these settlements, but also has a certain restrictive effect on its spatial layout [29]. The landform of the settlement areas are mainly mountainous, with small plains and hills. The north is mainly high mountains, with an average altitude of 1200m. The middle, south and east are low mountains and hills, with 200m-500m. In the west, there are low mountains and middle mountains, with 500m to 1000m. The settlements have mainly concentrated in the low mountains and hills areas, and 81.13% of them distributed below 1000m (Fig 10A). Terrain elements can also be quantified by RDLS, and GIS was used for raster calculation of Formula (4) to obtain the spatial distribution map of RDLS in Wuling Mountains area (Fig 10B), thus counted the percentage of settlements in each range with different RDLS values (Table 4). The results reveal that larger RDLS is located in the north and northeast of the region, and lesser in the east and south. In general, the relief degree in the area varies gently, which is low-relief terrain, that is mountains with even height. When its value is 0–1.5, a larger number of settlements distributed, with a cumulative frequency of 98.11%, among which settlements are more concentrated 0.5–1.0, accounting for 58.87%. The conclusion shows that the Tujia traditional settlements mainly live in the low-relief terrain with gentle elevation. With the spatial distribution density of settlements gradually decreases as the RDLS value increases, and the settlements are mostly concentrated along the Wuling Mountains. Therefore, it can be inferred that the ancestors chose the flat terrain for the purpose of farming.

**Table 4:** RDLS in each region and the percentage of traditional Tujia settlements.

Settlement areas	Percentage of settlements in each RDLS region					
	0–0.5	0.5–1.0	1.0–1.5	1.5–2.0	2.0–2.5	2.5–3.0
Tujia settlement area	16.60%	58.87%	22.64%	1.89%	0.00%	0.00%

#### 5.2.2 Slope and aspect analysis

**Fig 11:**
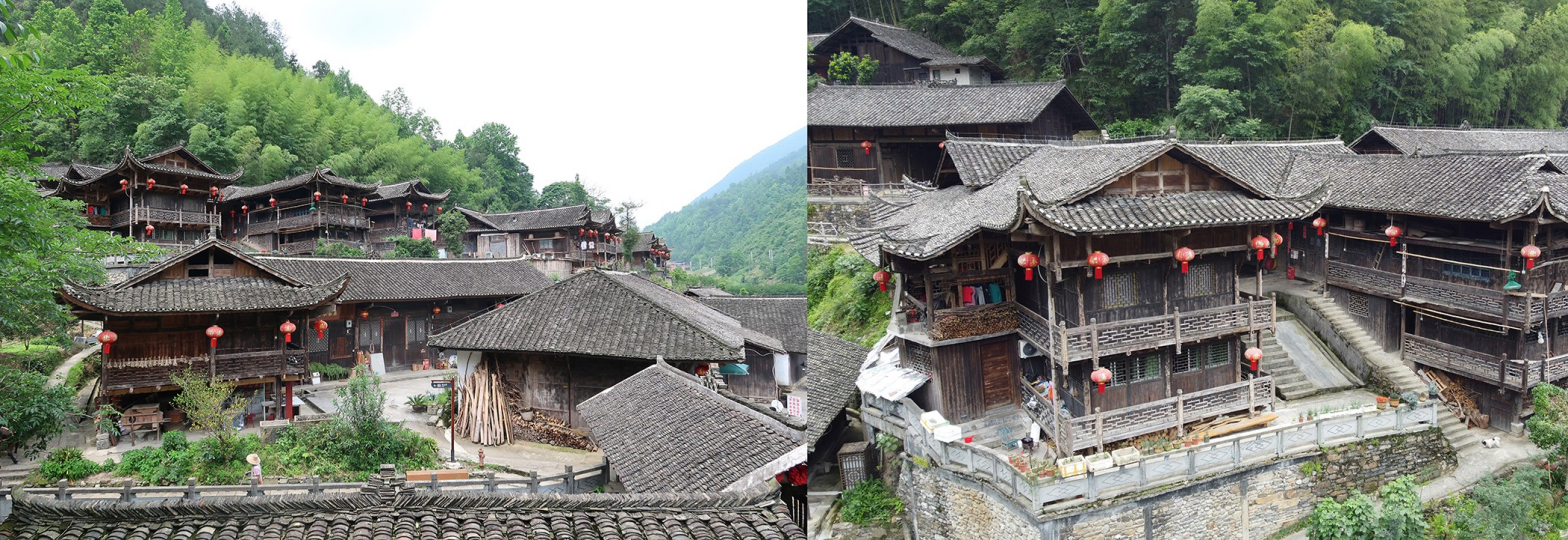


Most of the Tujia traditional settlements are located within the slope range of 5°-25°, with 172 sites and accounting for 61.3% of the total area. There are only 6 with a slope less than 5°, and 87 with a slope of 25°-45° (Fig 10C and Table 5). Although China’s current planning code describes land with slopes steeper than 25° as being unsuitable for building on [12], the Tujia ancestors constructed on slopes due to the special topography of the Wuling Mountains, and came into being with the construction of stilted houses (Fig 11), which were closely integrated with the mountain and made part or all of the bottom surface of the buildings completely separate from the surface. This architectural form did not change the original terrain characteristics and could fully adapt, making it the best choice for the construction of residential houses in the mountain terrain. Conducive to ventilation and avoid moisture. Further research found that the mountainous area lacked flat land, ancestors reserved flatter lands for farming, which reflected the fundamental principle of the settlement construction is to ensure that farmland is not occupied, and this’s the inevitable choice for the ancestors under severe natural conditions. The unique terrain has led to the construction of the wooden stilted houses, which also reflects the natives’ ecological wisdom.

**Table 5:** Slope of Tujia settlement regions.

Slope inclination	Number of settlements	Number percentage	Regions percentage
0–5°	6	2.3%	5.9%
5–25°	172	64.9%	61.3%
25–35°	49	18.5%	20.8%
35–45°	38	14.3%	9.0%
45–81.22°	0	0%	3.0%

Fig 10D shows Tujia traditional settlements are distributed in all slope directions (north, northeast, east, southeast, south, southwest, west and northwest). According to the category of sunny slope (157.5°-337.5°) and shady slope (0°-157.5°, 337.5°-360°), the ratio is about 1.3: 1, indicating that there are settlements on both sides of the mountain. There is a close relationship between the slope aspect and sunshine. The settlements located on the sunny slope have abundant sunshine, while located on the shady slope tend to rely on the low hills and do not lack enough sunshine not because the shelter of the mountain. There are fewer settlements on the steeper shady slopes where sunshine is lacking. It shows that the slope aspect has a certain impact on the distribution of settlements, and sunny slope with sufficient sunshine is the primary choice, while choice shady slope can obtain sufficient sunshine through site selection and construction strategies adapted to local conditions.

#### 5.2.3 Climatic and hydrological analysis

The Wuling Mountains have a monsoon-influenced humid subtropical climate, with warm and humid weather, abundant precipitation, and the average annual precipitation is 1068.7mm-1635.5mm (Fig 10E), with about 180 rainy days a year. And the average annual temperature is 5°-16°, with no severe temperature in summer or winter, and extensive river coverage. Which all have a certain impact on settlement site selection and construction. Overlay the river data with settlements to generate the distribution map of settlements and the river system (Fig 10F). Statistics show that most settlements were built along river, accounting for more than 90%, and 53.9% of settlements were located along the small rivers (river width ≤ 20m). The analysis shows that it’s a common feature for Tujia traditional settlements to be built along the river, with rely on but not close to the river, and presenting a pattern of “living by the mountains and near the water”. Which not only ensures sufficient water supply but also dry settlement and free from the threat of flooding. It has a strong ability to adapt to the natural environment and has its unique construction techniques in architectural.

### 5.3 Historical evolution of settlements distribution

**Fig 12:**
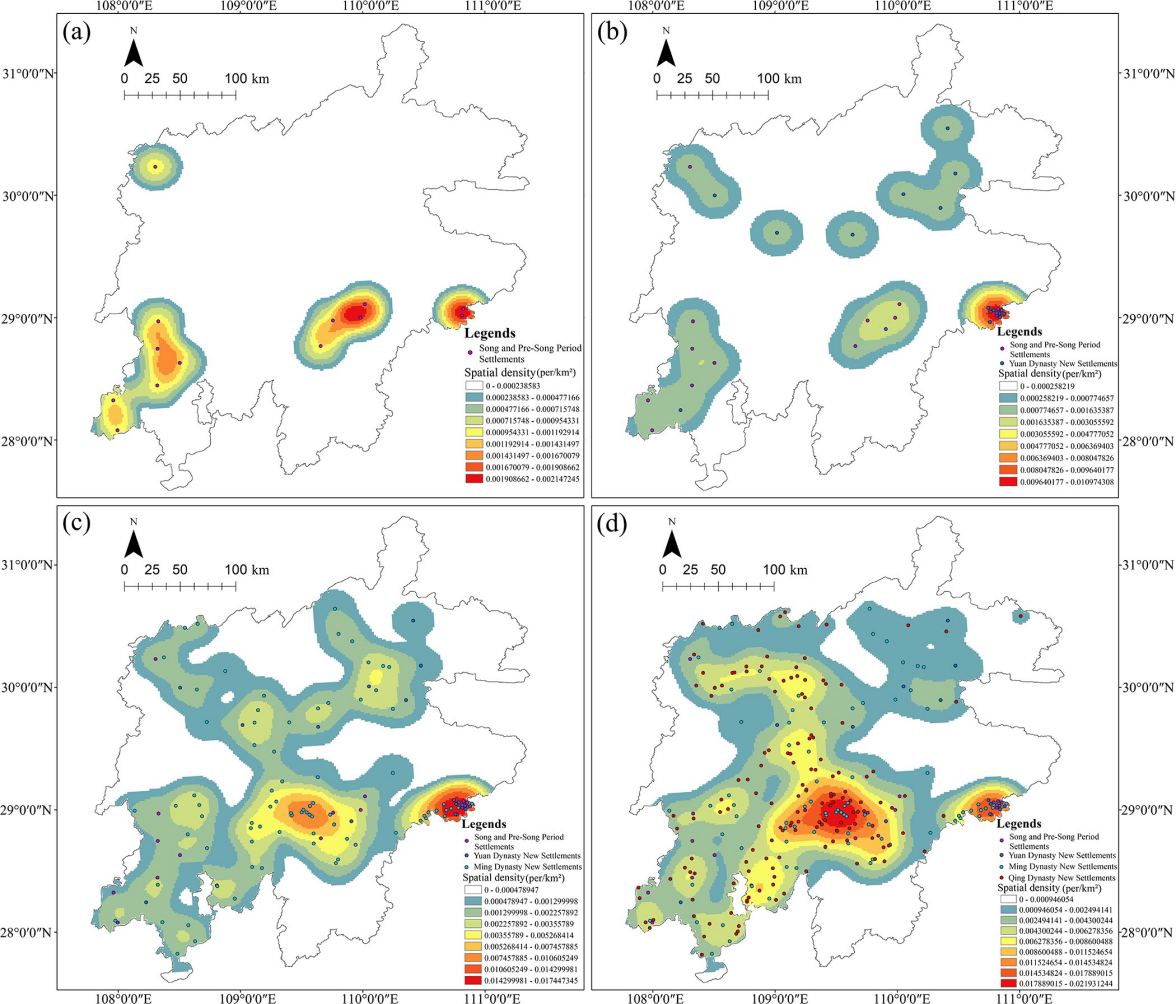


With a long history, the development of the Tujia ethnic minority can be traced back to the Shang and Zhou dynasties. During that period, people in the Central Plains of China called the Tujia ancestors the “Ba people”. Since the Qin Dynasty, the ethnic minorities settled on the borders of Hunan, Hubei, Guizhou provinces, and Chongqing municipality, were commonly known as “Man (barbarians)”. During the Song Dynasty, the term “Miao” was introduced, and the terms “Tu Ding”, “Tu Min” and “Tu Man” appeared one after another, referring specifically to the Tujia people in western Hubei and Hunan. And during the late Qing Dynasty, the term “Tujia” appeared in some local chronicles. The migration process of the Tujia minority in different periods is as follows: Xia Dynasty (the “Ba people” in Shaanxi and Gansu provinces), Shang Dynasty (Shangqiu in Henan Province, south of the Weishui River), Zhou Dynasty (Hanshui River basin), Spring and Autumn and Warring States period (eastern Sichuan Province, Langzhong), Qin and Han dynasties (Qingjiang River basin, southwest of Hubei Province), after Qin and Han dynasties (the Tujia minority in Wuling Mountains) [16, 28]. Most of the Tujia settlements left today were built during the Song, Yuan, Ming, and Qing dynasties, and the distribution and evolution of these settlements during four periods are shown in Fig 12. To better present the chronological development, the ANN analysis was conducted (Table 3). The results indicate that Tujia traditional settlements show significant clustering in different periods, but exist some differences. In general, from the Song to the Qing Dynasty, there were ups and downs in the degree of clustering. Three periods of changes are discussed below: from the Song to the Yuan and the Ming to the Qing Dynasty, the degree of clustering tended to gradually decrease, while the degree of the latter was greater than that in the former. On the contrary, from the Yuan to the Ming Dynasty, the degree tended to increase, and the settlement regions evolved with the changes of the settlements in each period.

During the Song Dynasty, the settlements were mainly located in the southern and western Wuling Mountains (Fig 12A), and the regions were so sparsely populated that “the wealthy and powerful encouraged others to move their families there” [16]. To effectively control the Tujia areas, the Song government set up military fortresses. And according to the Song History Geography Monograph, there were more than 80 fortresses in the regions of Chenzhou, Shizhou, Sizhou, mainly set up in the south of Wuling Mountains, meanwhile, many Han Chinese were integrated into the Tujia as well. In the Yuan Dynasty, settlements began to appear in the north of Wuling Mountains (Fig 12B), with a few new settlements and a decrease in the degree of clustering. At one time during that period, a defense system was introduced in Wuling Mountains, and the civilians were selected to work for military agro-colonies. These civil colonies and fortresses are some of the remaining settlements.

During the Ming and Qing dynasties, the number of settlements increased over time, and the degree of clustering was greater. Particularly during the Ming Dynasty, has the highest level of clustering, but the number of new settlements was less than that of the Qing Dynasty. The settlements were mainly clustered in the central part of the Wuling Mountains (Fig 12C). During this period, there was an enormous migration movement of “Huguang Filling Sichuan” (Huguang roughly encompassed today’s provinces of Hubei, Hunan, Guangxi, and parts of Guangdong and Guizhou provinces), and when these immigrants traveled through the Wuling Mountains, most of them stayed and merged with the Tujia people, so the degree of clustering was highest. The largest number of new settlements was recorded during the Qing Dynasty, and this is relevant to the construction of the chieftain resident [16] and the transportation and marketing of Sichuan salt [17]. From the Yuan to the early Qing Dynasty, the feudal government set up the chieftain system in the Tujia regions, establishing numerous chieftains resident (Fig 8), it was stipulated that “native residents were not allowed to leave the territory and no Han Chinese were permitted to enter” [28] to ensure the integrity of the Tujia ethnic group. Then, settlements were built around the residents. The transportation and marketing of Sichuan salt developed during the Ming and Qing dynasties, covering mainly Wuling Mountains area. As remote and inaccessible as the Wuling Mountains were, besides shipping by river, salt was mostly transported by humans and horses, so many bazaars and post stations flourished along the route, and then a large number of ancient residential settlements were built with local architectural style. During the Yongzheng period of the Qing Dynasty, the government weakened the power of chieftains and implemented the policy of “bureaucratization of native officers”, returning the rights of land to the peasants. Thus, the salt industry gradually recovered in the salt towns of Sichuan, leading to a boom in the marketing of Sichuan salt, and enabling the economic exchanges between the Tujia and other ethnic groups. Moreover, post stations and marketplaces along the routes of Sichuan salt marketing in the Wuling Mountains emerged and gradually expanded, allowing the development of settlements, so that the largest number of settlements appeared in that period (Fig 12D). The Tujia traditional settlements which remain are mostly located near the main land routes for the transportation and sale of Sichuan salt.

## 6. Conclusions

Most previous studies have analyzed the characteristics of Tujia traditional settlements at the provincial or smaller level, often overlooking the spatio-temporal heterogeneity of the distribution of Tujia settlements within in China. This paper has described and interpreted the spatial-temporal patterns and evolution of Tujia traditional settlements distribution. With the basic information of 265 traditional settlements, GIS technology and statistical analysis methods, several characteristic values affecting the distribution of settlements were calculated and a series of maps were created. The integration and analysis of natural environment and settlements datas as well as calculations, including kernel density estimation analysis, nearest-neighbor analysis and road accessibility analysis, have provided a broad long-term perspective on the spatial distribution and historical evolution of settlements, and then inspired and promoted the in-depth study of traditional settlements in other regions and other ethnic groups.

Two main conclusions are drawn from the results and discussions. First, in terms of space, there are significant spatial differences in the distribution of Tujia traditional settlements, with the overall patterns of “two belts, one core and multiple nodes”, and the average distribution density of 33 units per 10,000 km^2^, showing the characteristics of clustering. At the same time, the distribution of settlements is affected by elevation, RDLS, slope and aspect. And the settlements are mainly located in low mountains and hills, gentle slopes, sunny slopes and low-relief terrain areas, with the number of settlements gradually decreases as the RDLS value increases. And the overall road accessibility of settlements is good. Second, in terms of time, the spatial distribution of settlements exhibited a pattern of clustering from Song to Qing Dynasty. The Ming Dynasty witnessed the highest degree of settlements clustering, mainly in the northwest of Hunan, influenced by the large migration movement of “Huguang Filling Sichuan”. During the Qing Dynasty, when the transportation and marketing of Sichuan salt reached its peak, the Tujia settlements along the ancient routes emerged and gradually expanded, with the spatial distribution patterns of “scattered distribution in a large area, while concentrated in small areas”, and the settlement regions evolved along with the changes of these settlements in each period. However, the influence of natural or human environment on the settlement does not exist in isolation, they interweave with each other through specific forms, media and ways, and jointly determine the survival, and development of the settlement. Focusing on the task of village construction to promote rural revitalization, this study explores the spatio-temporal pattern and evolution of settlements distribution, which plays an important role in formulating centralized and contiguity protection measures for traditional Tujia settlements and promoting settlement protection and sustainable development.

### Notes

Ⅰ. Overlay analysis(OA) is a method of superposition two or more groups of factors in the same area to generate new features. In this paper, the geographic location information of settlements and spatial datas such as elevation, slope, aspect, river and ancient Sichuan salt road were overlaid analysis in GIS to analyze the relationship between settlement distribution and various elements, so as to reveal the distribution regular characteristics.
